# Overview of systematic reviews - a new type of study. Part II: Overview de revisões sistemáticas - um novo tipo de estudo. Parte II

**DOI:** 10.1590/1516-3180.2013.8150015

**Published:** 2014-11-07

**Authors:** Valter Silva, Antonio Jose Grande, Alan Pedrosa Viegas de Carvalho, Ana Luiza Cabrera Martimbianco, Rachel Riera

**Affiliations:** I BSc. Specialist in Cardiac Rehabilitation, Obesity and Statistics and Doctoral Student in the Postgraduate Program on Internal Medicine and Therapeutics, Escola Paulista de Medicina, Universidade Federal de São Paulo (EPM-Unifesp), São Paulo, Brazil.; II BSc, MSc. Doctoral Student in the Postgraduate Program on Internal Medicine and Therapeutics, Escola Paulista de Medicina, Universidade Federal de São Paulo (EPM-Unifesp); Volunteer Research Assistant at the Brazilian Cochrane Center, São Paulo, Brazil.; III BSc, MSc. Specialist in Rehabilitation and Cardiac Physiotherapy and Doctoral Student in the Postgraduate Program on Internal Medicine and Therapeutics, Escola Paulista de Medicina, Universidade Federal de São Paulo (EPM-Unifesp); Volunteer Research Assistant at the Brazilian Cochrane Center, São Paulo, Brazil.; IV BSc. Specialist in Orthopedics and Doctoral Student in the Postgraduate Program on Internal Medicine and Therapeutics, Escola Paulista de Medicina, Universidade Federal de São Paulo (EPM-Unifesp); Volunteer Research Assistant at the Brazilian Cochrane Center and Preceptor at EPM-Unifesp, São Paulo, Brazil.; V MD, MSc, PhD. Rheumatologist and Professor at Escola Paulista de Medicina, Universidade Federal de São Paulo (EPM-Unifesp); Coordinator at Brazilian Cochrane Center, São Paulo, Brazil.

**Keywords:** Review [publication type], Study characteristics [publication type], Decision making, Evidence-based practice, Evidence-based medicine, Revisão, Características dos estudos, Tomada de decisões, Prática clínica baseada em evidências, Medicina baseada em evidências

## Abstract

**CONTEXT AND OBJECTIVE:**

Overviews of Systematic Reviews (OoRs) are a new type of study in which multiple evidence from systematic reviews (SRs) is compiled into an accessible and useful document. The aim here was to describe the state of the art and critically assess Cochrane OoRs that have been published.

**DESIGN AND SETTING:**

Descriptive study conducted at a research center.

**METHODS:**

The OoRs identified through the filter developed in Part I of this study were evaluated in five domains: methodological quality; quality of evidence; implications for practice; general profile of OoRs; and length of work.

**RESULTS:**

All 13 OoRs included had high methodological quality. Some OoRs did not present sufficient data to judge the quality of evidence; using sensitivity analysis, the quality of evidence of the OoRs increased. Regarding implications for practice, 64% of the interventions were judged as beneficial or harmful, while 36% of them showed insufficient evidence for judgment. It is expected (with 95% confidence interval) that one OoR will include 9,462 to 64,469 patients, 9 to 29 systematic reviews and 80 to 344 primary studies, and assess 6 to 21 interventions; and that 50 to 92% of OoRs will produce meta-analysis. The OoRs generated 2 to 26 meta-analyses over a period of 18 to 31 months.

**CONCLUSION:**

The OoRs presented high methodological quality; the quality of evidence tended to be moderate/high; most interventions were judged to be beneficial/harmful; the mean length of work was 24 months. The OoR profile adds power to decision-making.

## RESUMO

**CONTEXTO E OBJETIVO:**

*Overviews* de revisões sistemáticas (OoRs) representam um novo tipo de estudo que compila múltiplas evidências de revisões sistemáticas (SRs) em um documento acessível e útil. O objetivo foi de descrever o estado da arte e avaliar criticamente as OoRs Cochrane publicadas.

**DESENHO E LOCAL:**

Estudo descritivo realizado em centro de pesquisa.

**MÉTODOS:**

As OoRs identificadas através do filtro desenvolvido na parte I deste estudo foram avaliadas por cinco domínios: qualidade metodológica, qualidade da evidência, implicações para a prática, perfil geral das OoRs e tempo de execução.

**RESULTADOS:**

As 13 OoRs incluídas apresentaram alta qualidade metodológica. Algumas OoRs não apresentavam dados suficientes para julgar a qualidade da evidência; com a análise de sensibilidade, a qualidade evidência nas OoRs aumentou. Implicações para prática foram julgadas como benéficas ou danosas em 64% das intervenções; em 36% das intervenções há evidências insuficientes para o julgamento. É esperado (com intervalo de confiança de 95%) que uma OoR inclua 9.462 a 64.469 pacientes, 9 a 29 revisões sistemáticas e 80 a 344 estudos primários, e avaliem entre 6 e 21 intervenções; que 50 a 92% das OoRs realizem metanálise. As OoRs geraram entre 2 e 26 metanálises em um período de 18 a 31 meses.

**CONCLUSÃO:**

As OoRs apresentam alta qualidade metodológica; a qualidade da evidência tende a ser alta/moderada; as intervenções, em sua maioria, foram julgadas como benéficas/danosas; o tempo de execução foi de 24 meses em média. O perfil das OoRs potencializa a tomada de decisão.

## INTRODUCTION

Overviews of Systematic Reviews (OoRs) are a new type of study that has been proposed by the Cochrane Collaboration in order to compile multiple evidence from systematic reviews (SRs) into a single document that is accessible and useful. Each OoR focuses on a problem or medical condition for which two or more SRs have addressed potential interventions and their outcomes.^1^^,^^2^^,^^3^^,^^4^^,^^5^^,^^6^

One SR rarely addresses all potential interventions for a condition, and healthcare policymakers may have difficulty in finding, evaluating, comparing and summarizing the information from all the relevant SRs.^3^^,^^4^ Thus, the main objective of OoRs is to serve as a friendly front end for the Cochrane Collaboration with regard to healthcare decision-making. The relevant SRs are integrated and/or summarized into a single document, i.e. an OoR. This, in theory, allows the reader to have access to an integrated summary of a long list of studies included in Cochrane SRs.^1^^,^^2^^,^^3^^,^^4^^,^^5^^,^^6^ Therefore, the primary audience for OoRs are healthcare decision-makers, such as healthcare professionals, policymakers and informed consumers who, through the Cochrane Library, seek evidence on treatments for various health conditions.^1^^,^^2^

The first part^1^^,^^2^ of this series of three articles on OoRs focused on the growth of publications with the best level of evidence available for healthcare decision-making. It provided justifications for implementing this new type of study, as well as defining who the target audience are. Furthermore, a filter was created and applied in order to search for specific OoRs in the Cochrane Library.

This second part of the series continues to address this topic by describing the state of the art (state of knowledge) of Cochrane Collaboration OoRs, through a critical assessment of Cochrane OoRs. In Part III, a new hierarchy for the pyramid of evidence will be proposed, taking this new type of study into consideration.

## OBJECTIVE

To critically assess Cochrane Overviews of Systematic Reviews, through analyzing the characteristics, approaches and methodological aspects of this type of study.

## METHODS

This descriptive study was conducted at the research center of a federal university in Brazil and within one of its postgraduate study programs.

**Figure 1.**
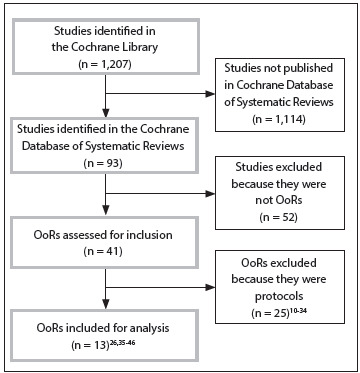
Preferred Reporting Items for Systematic Reviews and Meta-Analysis flowchart for Cochrane Overviews of Systematic Reviews (OoRs).

We performed a search for OoRs in the Cochrane Library, as described in Part I^1^^,^^2^ of this series. The flowchart for the OoRs is shown in Figure 1. The inclusion criteria were that the studies needed to be OoRs and to have been published in the Cochrane Database of Systematic Reviews, which is one of the six directories of the Cochrane Library.^7^ Protocols for Cochrane OoRs that have been published were excluded.

After OoR selection, two of the present authors (VS and AJG) read them, extracted data and assessed the quality. Differences in data collection information were resolved by reaching a consensus.

The data extracted from each OoR were organized using a specific form that sought information on the research question and objective, date of search, number of studies included, participants, interventions, main outcome, methodological quality of the review, quality of evidence and authors’ conclusion.

We used five items to critically assess the OoRs: (1) methodological quality, using the AMSTAR tool (Assessing the Methodological Quality of Systematic Reviews*)*;^8^ (2) quality of evidence, assessed using the GRADE tool (Grades of Recommendation, Assessment, Development and Evaluation) or any other method reported through OoRs; (3) implications for practice (the evidence confirms that the intervention presents benefits; the evidence confirms that the intervention presents harm/risk; or absence of evidence for a recommendation); (4) general profile of OoRs included (patients/OoR, SRs/OoR, studies/OoR, interventions/OoR, meta-analysis/OoR and search strategy); (5) Length of work, i.e. the time taken to publish the OoR (in years), obtained from the date of registration of the title (via Archie), date of publication of the protocol (via the Cochrane Library) and date of publication of the OoR (via the Cochrane Library).

Data synthesis was performed using descriptive statistics. Contingency tables were used to summarize dichotomous data as frequencies and proportions. Quantitative data were summarized using the mean and standard deviation. Sensitivity analysis were performed to assess the robustness of the results.

## RESULTS

The search filter for OoRs that was developed in Part I^1^^,^^2^ of this study was updated, validated^9^ [sensitivity = 1.00 (95% CI = 0.86 to 1.00); specificity = 0.99 (95% CI = 0.99 to 1.00)] and used (on November 5, 2013). Through this process, 1207 titles were retrieved, of which 95% were excluded because they had not been published in the Cochrane Database of Systematic Reviews. Ninety-three references were checked, but 52 of these were systematic reviews and were excluded; and another 26 potential OoRs^10^^,^^11^^,^^12^^,^^13^^,^^14^^,^^15^^,^^16^^,^^17^^,^^18^^,^^19^^,^^20^^,^^21^^,^^22^^,^^23^^,^^24^^,^^25^^,^^25^^,^^27^^,^^28^^,^^29^^,^^30^^,^^31^^,^^32^^,^^33^^,^^34^^,^^35^ were excluded because they were at the protocol stage. At the end of the selection process, 13 OoRs^26^^,^^35^^,^^36^^,^^37^^,^^38^^,^^39^^,^^40^^,^^41^^,^^42^^,^^43^^,^^44^^,^^45^^,^^46^ were included, were used for data extraction and were critically assessed. Figure 1 presents the PRISMA (Preferred Reporting Items for Systematic Reviews and Meta-Analysis) flowchart for the OoRs.

The characteristics of the thirteen OoRs included in this study are described in detail in Appendix 1, which includes the main conclusions from the approaches used in each study. The synthesis on the data extracted from these 13 OoRs, with the overall assessment, is presented in Table 1.

**Table 1.**
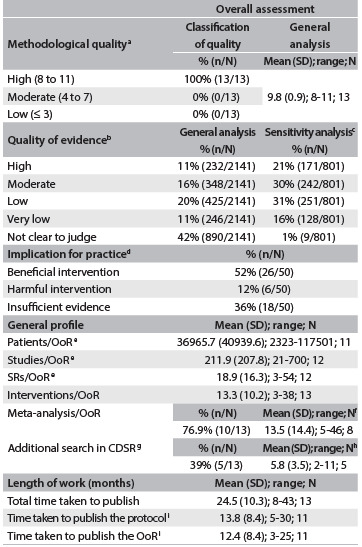
Overall assessment of Cochrane Overviews of Systematic Review (OoRs)

^a^AMSTAR conducted;^8 b^frequency based on the number of outcomes or studies assessed; ^c^OoRs^41,43,48^ in which more than 10% of the studies/categories could not be clearly judged regarding the quality of the evidence were excluded from the sensitivity analysis; ^d^judgments based on main outcome conclusion; ^e^some data could not be determined (patients/OoR,^39,41^ SRs/OoR^42^ and studies/OoR^41^), even by looking for results tables in the OoRs or SRs included, thus reducing the number of OoRs in the analysis; ^f^studies in which no meta-analysis was conducted^37,41,43-45^ were not included; ^g^CDSR is the abbreviation for Cochrane Database of Systematic Reviews; ^h^only OoRs^39,41-43,46^ in which additional searches in the CDSR were conducted were included in the analysis; ^i^the protocols for these OoRs^37,39^ were not found in the Cochrane Library or in Archie, thus reducing the number of OoRs in the analysis; SD = standard deviation; range = minimum to maximum;

All the OoRs that were included had high methodological quality, reaching between 9 and 11 points out of the 11 points possible in AMSTAR (Table 1). However, some OoRs lost points regarding methodological quality for the following reasons: they did not record the protocol^36^ or, if it was cited, did not make it available;^38^ they only included searches in the Cochrane Database of Systematic Reviews (CDSR); they did not consider the quality of evidence in formulating conclusions;^46^ they were unable to “meta-analyze” the data;^40^^,^^42^^,^^43^^,^^46^ and they did not assess the risk of bias among the reviews included.^36^

With regard to judging the quality of evidence, it was found that many categories/studies were unclear, and thus the proportion of the categories/studies that presented high-quality evidence was low. If the OoRs^40^^,^^42^^,^^46^ in which more than 10% of the studies/categories could not be clearly judged regarding the quality of evidence were excluded, the proportion of studies/categories that were of high quality doubled. The sensitivity analysis drastically altered the proportions of the other categories regarding the quality of evidence (Table 1).

Through the outcome of implications for practice, we found that about 64% of the interventions were judged to be beneficial or harmful. However, there was insufficient evidence to judge the interventions in 36% of them (Table 1).

Regarding general factors, with a 95% confidence interval, we expect that each new OoR will include between 9,462 and 64,469 patients, between 9 and 29 SRs and between 80 and 344 primary studies, and will assess between 6 and 21 interventions. Between 50 and 92% of OoRs that pool data qualitatively will generate between 2 and 26 meta-analyses. Additional details are analyzed in Table 1.

Most of the OoRs (62%) conducted searches for systematic reviews only in the Cochrane Database of Systematic Reviews (CDSR). However, those that conducted external searches, in addition to searching in the CDSR, used between one and ten databases (Table 1).

Table 1 also shows that, from the date when the OoR title was registered, the authors spent about two years (about one year for planning/preparing the protocol and another year for implementing the full study) until the date of publication.

Only 23% (3/13) of the OoRs did not identify the study type in the title. The same also occurred in the protocols for OoRs (6/28).

## DISCUSSION

The Cochrane Collaboration’s OoRs are supervised by one of the 16 methods groups, the Comparing Multiple Interventions Methods Group. This methods group of the Cochrane Collaboration was established in 2004 and initially was called the Umbrella Reviews Working Group.^47^

Other milestones during the development of the OoRs methodology occurred in 1996, 1997, 1998 and 2005. Between February 1997 and December 1998, the first series of OoRs,^48^^,^^49^^,^^50^^,^^51^ four reviews relating to pregnancy were published as a partnership between researchers from the UK Cochrane Centre and the World Health Organization. In 1996, Julian Higgins and Anne Whitehead published the first article to describe the standard Bayesian approach towards multiple-treatment meta-analysis (MTM).^52^ In 2005, in the 13^th^ Cochrane Colloquium in Melbourne, Georgia Salanti made a presentation on MTM methodology,^53^ co-authored with Julian Higgins and Valeria Marinho. This won the prize for best oral presentation and helped to popularize the technique.

In this context, the present systematic review describes how far the knowledge of the select group of Cochrane OoRs published in the CDSR has reached. Although we only included 13 OoRs, these studies published in the CDSR by the Cochrane Collaboration stand out through their methodological rigor. This was highlighted in two studies^54^^,^^55^ that compared the methodological rigor of Cochrane systematic reviews with non-Cochrane systematic reviews. On January 24, 2011, in recognition of the contribution to healthcare that the Cochrane Collaboration has made,^56^ the World Health Organization lauded the Collaboration as a non-governmental organization, with a seat in the World Health Assembly and voting rights, in order to help manage healthcare worldwide.

As examples of Cochrane systematic reviews,^54^^,^^55^ all the OoRs included in this review had high methodological quality and therefore less risk of bias, according to the results obtained through AMSTAR.^8^ However, the reasons why not all the OoRs included obtained the maximum score need to be discussed:

1. The protocols for two OoRs^36^^,^^38^ were not found in the Cochrane Library or in Archie, even though one of them cited the protocol.^38^ Registration of the protocols for OoR, just as for clinical trials and systematic reviews, allows assessment of methodological quality, provides transparency in conducting the study and minimizes occurrences of publication bias or selective reporting of outcomes.
2. Eight OoRs restricted the search to only one database, i.e. the CDSR. Moreover, we did not consider this to be a potential source of bias, taking the view that the search methods for systematic reviews in the CDSR are rigorous and comprehensive (including no restrictions on date, publication status or language), and therefore that there was a high probability of including all the relevant primary studies.
3. Four OoRs could not match any of the systematic reviews included in their quantitative data synthesis, through either direct or indirect meta-analysis, and therefore summarized and integrated the evidence qualitatively.
4. One OoR^36^ did not evaluate or discuss the risk of publication bias among the systematic reviews included.

Although AMSTAR^8^ was developed to assess the methodological quality of systematic reviews, it needs to be borne in mind that many of the items that it assesses are also present in OoRs, even though OoRs are different studies. Thus, AMSTAR^8^ can be considered to be an analogous tool for assessing OoRs, in which external validity is preserved. Development of an instrument for judging the methodological rigor and consistency of OoRs will be important for reducing the uncertainties in decision-making from these studies.

One challenge identified in the present study was the lack of standardization in the method for assessing the quality of evidence. OoRs have used various methods of assessment (e.g. GRADE,^57^ Cochrane risk of bias tool,^58^^,^^59^ Jadad et al.,^60^ or even review-specific criteria) or, when using the same method, have described it in different ways, thereby making the judgment uncertain. In this context, two OoRs^40^^,^^42^ can be highlighted. Despite the high methodological quality of the study by Ryan et al.,^40^ it only described how the systematic reviews included had assessed the quality of evidence, thus making it difficult to assess this outcome. Many studies (890/2141) may not have made judgments regarding the quality of evidence. In the second of these OoRs, Jones et al.^42^ used the Cochrane risk of bias tool^58^^,^^61^ to assess the quality of the evidence in Cochrane reviews, while for non-Cochrane reviews they used Jadad et al.^60^ Despite the differences in the concepts of quality of evidence and methodological quality (which were not among the objectives of this discussion), these authors reported the proportion of studies that presented high quality. Thus, the description was not sufficiently clear or standardized for a judgment to be made regarding the overall quality of evidence.

To assess the quality of evidence, the most consistently used method among the OoRs assessed was the GRADE approach.^57^ Basically, this tool classifies evidence into four levels: (1) high quality - it is unlikely that future research will change the estimated effect; (2) moderate quality - future research may have a major impact on the estimated effect and change the result; (3) low quality - further research is very likely to have an important impact on the estimated effect and change the results; and (4) very low quality - there is no certainty in the estimated effect. In the GRADE approach to the classification of evidence, the assessment can be lowered or raised according to certain methodological factors. Methodological limitations, inconsistent results, imputations of evidence, imprecision of results and publication bias diminish the quality of evidence. The magnitude of the effect, controlling for confounders and dose-response gradients are methodological factors that may raise the level of evidence.

Even though there has been criticism regarding the lack of methodological rigor in this type of study,^62^^,^^63^ all the Cochrane OoRs assessed methodological quality, thus allowing judgment of the effects of interventions based on the main outcomes found. About two-thirds of the OoRs presented evidence to judge how beneficial or harmful the main outcomes were. These results are based on various systematic reviews and thus integrate and summarize evidence from several interventions relating to a given condition, from a high number of clinical trials and patients. The evidence in these OoRs was released over a period of about two years from the time of registration of the title to the date of publication. In the future, it will be important to also determine the time taken to update the OoRs.

Furthermore, comparing the results from this descriptive systematic review of published studies with other OoRs^62^^,^^63^ that assessed this new type of study, the Cochrane OoRs seem to have higher quality and methodological rigor than non-Cochrane OoRs. To reduce the inconsistency of OoRs, it is important that authors follow the existing methodological recommendations.^3^^,^^5^^,^^61^

When extracting data, attention was drawn to two factors: (1) three studies^37^^,^^39^^,^^43^ did not declare the type of study, i.e. “overview of reviews” (there was a similar proportion with regard to protocols for OoRs: 6/28), which may be a limiting factor in that it becomes more difficult for readers to easily identify the type of study; (2) Singh et al.^41^ only included primary studies from the list of references of systematic reviews and conducted search strategies in bibliographic databases.

Another feature that may pose a challenge in conducting this type of study is the complex statistical methods required for integrate and summarizing the evidence. The methodological basis for network meta-analysis, also known as multiple-treatment meta-analysis and mixed-treatment comparisons, was established in 1996.^52^ In view of the challenge of implementing these statistical techniques, a special issue of Research Synthesis Methods was published on this topic in 2012 (see volume 3, number 2). For researchers who wish to conduct an OoR, it is highly recommended that the information provided by the Cochrane Comparing Multiple Interventions Methods Group, the Multiple-Treatment Meta-analysis website <http://www.mtm.uoi.gr/> and the special issue of Research Synthesis Methods should be consulted.

This new type of study certainly attracts everyone’s attention, as demonstrated from data in Evidence-Based Child Health.^5^ In 2009, OoRs were downloaded 3.05 times more than other types of study. There has also been a tendency towards growth of this frequency over the years (2006 versus 2009), by a rate of 1.84 times. The importance of this type of study can be understood through the great interest of its audience.

Despite the limitation of the small number of OoRs included and the absence of some information that could not be obtained, it could be seen that this type of study presents the following potential benefits: (1) it allows consumers to know about and understand the concepts and application of such studies and their ability to integrate and summarize various interventions for a disease or health condition; (2) for healthcare policymakers, summarizing all interventions into a single document allows them to keep up to date in the era of information globalization^1^^,^^2^ and, thus, this type of study serves as a friendly front end for healthcare decision-making based on the best available evidence, thereby changing professional practice and healthcare policies; and (3) it allows researchers to address the gaps, weaknesses and strengths of each study and think about future research strategies based on what still needs to be explored.

In Part III, a new hierarchy for the pyramid of evidence will be proposed, taking this new type of study into consideration.

## CONCLUSION

OoRs have high methodological quality and high quality of evidence, as assessed in general. Moreover, about two-thirds of the conclusions had sufficient evidence to judge that the implications for practice were either beneficial or harmful. In order to do this, the mean length of work required was 24 months.

The profile of OoRs reduces uncertainty in decision-making. This new type of study demonstrates the ability to compile multiple evidence from systematic reviews in one handy and useful document that is able to address all potential interventions for a condition, thereby allowing decision-makers to locate, evaluate, compare and summarize the evidence from systematic reviews or primary studies.

Although OoRs are able to integrate and summarize multiple interventions for a problem in a single document, there is still a need to standardize the methods for this new type of study.

## Figures and Tables

**Appendix 1.**
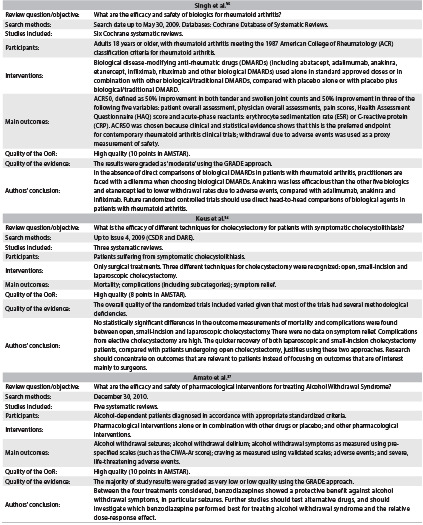
Characteristics of Cochrane Overviews of Systematic Reviews (OoRs) that were included.

**Appendix 1.**
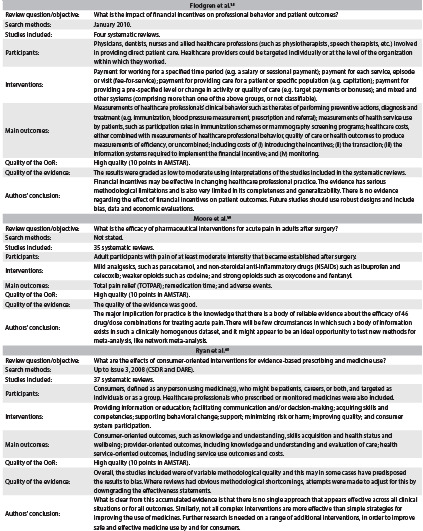
Continuation

**Appendix 1.**
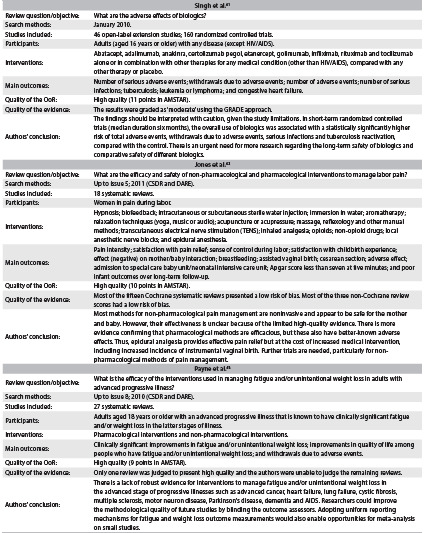
Continuation

**Appendix 1.**
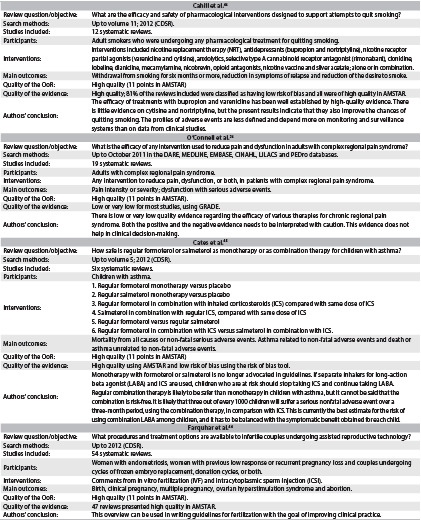
Continuation

GRADE = Grading of Recommendations Assessment, Development, and Evaluation; AMSTAR = A Measurement Tool to Assess Systematic Reviews; DARE = Database of Abstracts of Reviews of Effects.
